# The influence of high glucose on the aerobic metabolism of endothelial EA.hy926 cells

**DOI:** 10.1007/s00424-012-1156-1

**Published:** 2012-09-30

**Authors:** Agnieszka Koziel, Andrzej Woyda-Ploszczyca, Anna Kicinska, Wieslawa Jarmuszkiewicz

**Affiliations:** Department of Bioenergetics, Adam Mickiewicz University, Umultowska 89, 61-614 Poznan, Poland

**Keywords:** Endothelial cells, Mitochondrial respiration, Uncoupling protein, Hyperglycaemia, Oxidative metabolism

## Abstract

The endothelium is considered to be relatively independent of the mitochondrial energy supply. The goals of this study were to examine mitochondrial respiratory functions in endothelial cells and isolated mitochondria and to assess the influence of chronic high glucose exposure on the aerobic metabolism of these cells. A procedure to isolate of bioenergetically active endothelial mitochondria was elaborated. Human umbilical vein endothelial cells (EA.hy926 line) were grown in medium containing either 5.5 or 25 mM glucose. The respiratory response to elevated glucose was observed in cells grown in 25 mM glucose for at least 6 days or longer. In EA.hy926 cells, growth in high glucose induced considerably lower mitochondrial respiration with glycolytic fuels, less pronounced with glutamine, and higher respiration with palmitate. The Crabtree effect was observed in both types of cells. High glucose conditions produced elevated levels of cellular Q10, increased ROS generation, increased hexokinase I, lactate dehydrogenase, acyl-CoA dehydrogenase, uncoupling protein 2 (UCP2), and superoxide dismutase 2 expression, and decreased E3-binding protein of pyruvate dehydrogenase expression. In isolated mitochondria, hyperglycaemia induced an increase in the oxidation of palmitoylcarnitine and glycerol-3-phosphate (lipid-derived fuels) and a decrease in the oxidation of pyruvate (a mitochondrial fuel); in addition, increased UCP2 activity was observed. Our results demonstrate that primarily glycolytic endothelial cells possess highly active mitochondria with a functioning energy-dissipating pathway (UCP2). High-glucose exposure induces a shift of the endothelial aerobic metabolism towards the oxidation of lipids and amino acids.

**Electronic supplementary material:**

The online version of this article (doi:10.1007/s00424-012-1156-1) contains supplementary material, which is available to authorized users.

## Introduction

Mitochondria are found in most human cells; however, the synthesis of ATP in endothelial cells occurs primarily via a glycolytic pathway. The relatively slight dependence of endothelial cells on oxidative phosphorylation has created the perception that mitochondria play no significant role in the endothelium and has thereby resulted in the neglect of their study in this context. However, several recent observations challenge this view by suggesting that mitochondria not only can contribute to ATP generation but also are centrally involved in maintaining the fine regulatory balance among mitochondrial calcium concentrations, reactive oxygen species (ROS) production, and NO [4, 5, 13]. The endothelial mitochondria may also function as sensors of alternations in the local environment (in particular, changes in the perfusate constituents) and contribute to the survival of endothelial cells under oxidative stress. Moreover, there is emerging evidence that mitochondrial ROS are important signalling molecules in vascular endothelial cells [30].

The findings that exposure to high glucose affects endothelial cells have been subject of many studies since the mid 1990s ([1, 11, 20, 22] and references therein). Endothelial mitochondria may play a central role in the development of many cardiovascular diseases. The oxidative stress induced by hyperglycaemia is an important aspect of diabetic vascular disease. It has been proposed that the production of mitochondrial ROS in response to chronic hyperglycaemia might be the key initiator for several mechanisms by which hyperglycaemia damages cells, including increased flux through the hexosamine and polyol pathways, the increased formation of advanced glycation end products, and the activation of protein kinase C [1, 9]. Cases of inadequately controlled diabetes are correlated with an essential shift in myocardial energy utilization towards the oxidation of free fatty acids [5]. The impact of this shift on the mitochondrial metabolism in endothelial cells has been largely disregarded because these cells are considered to be primarily glycolytic in nature. However, it has been suggested that in endothelial cells, the activation of AMP-activated protein kinase (AMPK), which is a fuel-sensing enzyme, promotes the oxidation of fatty acids as the source of ATP production and downregulates the dependence of these cells on glycolysis [3]. Therefore, the role of endothelial mitochondria as energy sources might become significant in response to metabolic disturbances that relate to hyperglycaemia.

Uncoupling proteins (UCPs) are members of the mitochondrial anion carrier protein family that are present in the mitochondrial inner membrane and mediate free fatty acid-activated, purine nucleotide-inhibited proton conductance [21]. UCPs are involved in the control of cellular energy balance and help prevent the production of ROS. It has been shown that the upregulation of mitochondrial UCP2 by AMPK in endothelial cells attenuates oxidative stress in diabetes [29].

Many questions must be addressed with respect to understanding the physiological role that mitochondria play in endothelial cells and the contribution of endothelial mitochondria to vascular function and disease. For instance, no previous studies have directly demonstrated that isolated endothelial mitochondria are efficient and highly coupled. In addition, the present work presents the first functional characteristics of UCP2 in isolated endothelial mitochondria. This investigation also sought to determine how the aerobic metabolism in EA.hy926 cells that are supplied with different reducing fuels is altered by long-term growth in glucose concentrations much above the physiological range. To address this issue, the mitochondrial functions were compared in endothelial cells cultured in medium with either a high (25 mM) or a normal (5.5 mM) glucose concentration.

## Materials and methods

### Cell culture

**Fig. 1:**
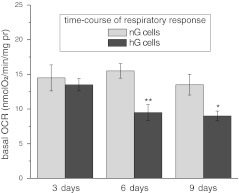
Time-course of cell respiratory response induced by excess glucose. EA.hy926 cells were cultured for 3, 6, and 9 days in normal-glucose concentration (nG cells) or high-glucose concentration (hG cells). The basal oxygen consumption rate (*OCR*) was measured with 5 mM pyruvate

A permanent human endothelial cell line EA.hy926 was originally derived from human umbilical vein and was kindly provided by Dr. Cora-Jean Edgell (University of North Carolina, NC, USA). Except for determination of a time-course of high-glucose-induced respiratory response (Fig. 1), the EA.hy926 cells were cultured for 6 days in DMEM culture medium with either 5.5 or 25 mM d-glucose (representing normal and high-glucose conditions, respectively). During cell culture the medium was changed after 3 days. This medium was supplemented with 10 % FBS, 1 % l-glutamine, 2 % HAT, and 1 % penicillin/streptomycin in a humidified atmosphere of 5 % CO_2_ at 37 °C. The EA.hy926 cells were cultured in dishes of 140 mm in diameter until they reached approximately 90–100 % confluence. Cells that were between passages 5 and 12 were used in this study.

### Measurements of cell respiration

The EA.hy926 cell cultures from 50 dishes (for both the normal and high-glucose cultures) were harvested with trypsin/EDTA, and rinsed twice with phosphate-buffered saline (PBS) (with 10 % and 5 % FBS, respectively), and centrifuged at 1,200×*g* for 10 min. Subsequently, the cells were washed in cold DMEJ medium containing 5.4 mM KCl, 0.8 mM MgSO_4_, 110 mM NaCl, 44 mM NaHCO_3_, 1.1 mM NaH_2_PO_4_, and 10 mM Na/Na buffer, pH 7.5, and were then centrifuged once again. The final cell pellet was resuspended in the same medium (1 g of cells per 2 ml medium) and kept on ice. The cells were counted prior to assays using a Burke haemocytometer. The total protein concentrations were determined using the Bradford method. The yield of harvested cells did not differ significantly between the normal and high-glucose cells as 3.32 ± 0.24 g of cells (805,000 ± 78,000 cells) and 3.77 ± 0.14 g of cells (870,000 ± 104,000 cells) (SE, *n* = 15), respectively, were harvested from 50 dishes of each culture. The cellular oxygen consumption rate (OCR) was measured at 37 °C using a Clark-type electrode (Hansatech) in 0.7 ml of DMEJ medium.

The mitochondrial function in detached EA.hy926 cells was determined polarographically as previously described for the extracellular flux analysis in adherent bovine aortic endothelial cells (BAECs) [6]. To estimate the ATP-linked OCR and non-ATP-linked OCR (proton leak) components of the basal respiratory rate, oligomycin (1 μg/ml) was added to inhibit ATP synthesis (Suplementary Data). Subsequently, the proton ionophore (uncoupler) carbonyl cyanide 4-(trifluoromethoxy) phenylhydrazone (FCCP, 0.4 μM) was added to determine the maximal OCR that the cells can sustain. Finally, cyanide (0.5 mM) was added to inhibit Complex IV (cytochrome *c* oxidase [COX]) and thereby block the entire mitochondrial cytochrome pathway. In the presence of cyanide, no residual (non-mitochondrial) respiration was observed.

### Nitroblue tetrazolium assay

ROS production was detected by nitroblue tetrazolium (NBT) assay [19]. NBT (yellow water soluble) was reduced by superoxide to formazan-NBT (dark-blue water insoluble). The assay was performed by incubating detached EA.hy926 cells (0.2 mg of protein in 1 ml DMEM medium with 5.5 or 25 mM glucose) with 0.2 % NBT under agitation. The samples were incubated for 1 h (37 °C) in the presence or absence of 10 μM diphenylene iodonium (DPI) (a NADPH oxidase inhibitor). The cells were centrifuged (1,200×*g* for 10 min at 4 °C), the supernatant was removed, and formazan-NBT was dissolved in 200 μl 50 % acetic acid by sonication (three pulses of 10 s; Bandelin Electronic). The samples were briefly centrifuged (spin down), and the absorbance of the supernatant was determined at 560 nm using a UV 1620 Shimadzu spectrophotometer.

### Mitochondria isolation

All of the subsequent steps were performed at 4 °C. After they were harvested and washed in PBS, EA.hy926 cells were resuspended in PREPI medium (0.25 M sucrose, 1.5 mM EDTA, 1.5 mM EGTA, 0.2 % BSA, and 15 mM Tris–HCl, pH 7.2) at a ratio of 3 ml medium per 1 g cells. The cells were then homogenised by ten passes with a tight Dounce homogeniser, and the homogenates were subsequently centrifuged at 1,200×*g* for 10 min. The pellets were resuspended, and the cells were once again homogenised (eight passes) and centrifuged to collect the mitochondria remaining in the pellet. The supernatants were combined and centrifuged at 1,200×*g* for 10 min, and the resultant supernatants were then centrifuged at 12,000×*g* for 10 min. The mitochondrial pellets were washed with a PREPII medium containing 0.25 M sucrose and 15 mM Tris–HCl, pH 7.2, and centrifuged at 12,000×*g* (10 min). The final pellet was resuspended in a small volume of the same medium. The yields of isolated mitochondria were equal to 4.2 ± 0.4 and 2.9 ± 0.4 mg mitochondrial protein/g cells (SE, *n* = 12) for cells grown in normal glucose and high glucose conditions, respectively.

### Measurements of mitochondrial respiration and membrane potential

Mitochondrial respiration and membrane potential (Δ*Ψ*) were measured as previously described [25]. Oxygen uptake was determined polarographically using a Rank Bros. (Cambridge, UK) oxygen electrode or a Hansatech oxygen electrode in either 1.4 ml or 2.8 ml of standard incubation medium (37 °C), which consisted of: 150 mM sucrose, 2.5 mM KH_2_PO_4_, 2 mM MgCl_2_, 20 mM Tris–HCl, pH 7.2, and 0.1 % BSA, with either 0.7 or 2 mg of mitochondrial protein. Δ*Ψ* was measured simultaneously with oxygen uptake using a tetraphenylphosphonium (TPP^+^)-specific electrode. The TPP^+^-electrode was calibrated by four sequential additions (0.4, 0.4, 0.8, and 1.6 μM) of TPP^+^. After each run, 0.5 μM FCCP was added to release TPP^+^ for baseline correction. For the calculation of the Δ*Ψ* value, the matrix volume of endothelial mitochondria was assumed to be 2.0 μl × mg^−1^ protein. The calculation assumes that the TPP^+^ distribution between mitochondria and medium followed the Nernst equation. The values of Δ*Ψ* were corrected for TPP^+^ binding using the apparent external and internal partition coefficients of TPP^+^ [28]. The correction shifted the calculated Δ*Ψ* values to lower values (approx. 30 mV shift), but it did not influence the changes in the resulting membrane potential (relative changes). The values of Δ*Ψ* are given in millivolts.

Phosphorylating respiration was measured using 150 μM ADP, and uncoupled respiration was measured using 0.25 μM FCCP. Non-phosphorylating (resting state) respiration measurements were performed in the absence of exogenous ADP and the presence of 1.8 μM carboxyatractyloside and 0.5 μg/ml oligomycin, which inhibited the activities of an ATP/ADP antiporter and ATP synthase, respectively. To induce UCP2 activity, palmitic acid (up to 21 μM) was used. To inhibit UCP2 activity, 2 mM GTP was applied. Proton leak assessments were performed as previously described [25].

The proton leak measurements were performed with 10 mM succinate (plus 1 μM rotenone) as an oxidisable substrate. The response of proton conductance to its driving force can be expressed as the relationship between the OCR and Δ*Ψ* (flux–force relationship) when varying the potential by titration with respiratory chain inhibitors. To decrease the rate of the *Q*-reducing pathway, succinate dehydrogenase was titrated with malonate (up to 5 mM).

### Measurements of the cellular and mitochondrial *Q*_10_ concentrations and the mitochondrial *Q* reduction level

The cellular and mitochondrial concentrations of coenzyme *Q*_10_ (*Q*) and the mitochondrial *Q* reduction level were determined by an extraction technique followed by HPLC detection as previously described [25]. A LiChrosorb RP*-*18 (10 μm) HPLC column was used for the separation of *Q*_10_. For the calibration and quantification of the *Q*_10_ peaks commercial coenzyme *Q* was used. The mitochondrial *Q* reduction levels are expressed as the percentage of total mitochondrial *Q* (QH_2_/*Q*_tot_).

### Measurement of mitochondrial enzyme activities

The activity of citrate synthase (CS) was determined by tracking the formation of DTNB-CoA at 412 nm [10]. The COX maximal activity was assessed with 2 mg cell protein or 0.25 mg mitochondrial protein without exogenously added respiratory substrate and in the presence of sequentially added antimycin A (10 μM), 8 mM ascorbate, 0.06 % cytochrome *c*, and up to 2 mM *N*,*N*,*N*′*N*′-tetramethyl-*p*-phenylenediamine (TMPD) (Fig. S1). The rate of oxygen consumption following the addition of TMPD reflected the maximal O_2_ consumption by COX (Complex IV). The estimation of outer mitochondrial membrane integrity was based on impermeability of the membrane to exogenous cytochrome *c*. A preparation induced damage of the outer mitochondrial membrane and as a result subsequent loss of cytochrome *c* can be detected by a stimulation of respiration (the COX activity) after the addition of cytochrome *c*. Thus, the percentage of the activity that is latent, i.e., hidden by a membrane, can be determined. Therefore, the integrity of outer membrane was assayed as the latency of COX activity during oxygen uptake measurements in the absence and presence of exogenous cytochrome *c* (Fig. S1). The same additions, i.e., 10 μM antimycin A, 8 mM ascorbate, 0.06 % cytochrome *c*, and up to 2 mM TMPD, were applied. No or slight acceleration of respiration by addition of exogenous cytochrome *c* prior to addition of TMPD indicated a high outer membrane integrity. The integrity was calculated from OCRs in the presence of given chemicals using the following equation: [TMPD_OCR_ − cytochrome *c*_OCR_]/[TMPD_OCR_ − ascorbate_OCR_]×100%.

### Determination of protein levels through immunoblotting

RIPA buffer (150 mM NaCl, 1 % Triton X-100, 0.5 % Na deoxycholate, 0.1 % SDS, 50 mM Tris, pH 8.0) was used to lyse the cells. The cellular and mitochondrial fractions were isolated in the presence of protease inhibitors (Sigma). The proteins were separated on a 10 % or 12 % SDS-PAGE gel. The spectra™ Multicolor Broad Range Protein Ladder (Fermentas) was used as a molecular weight marker. The following primary antibodies were used: mouse monoclonal anti-β actin (42 kDa) (CP01, Calbiochem), mouse monoclonal anti-hexokinase I (HK I, 120 kDa) (sc-80978, Santa Cruz Biotechnology), purified goat polyclonal anti-acyl-Coenzyme A dehydrogenase (ACADS, 44 kDa) (sc-107371, Santa Cruz Biotechnology), mouse monoclonal lactate dehydrogenase (LDH; 35 kDa) (sc-133123, Santa Cruz Biotechnology), purified goat polyclonal anti-E3-binding protein of pyruvate dehydrogenase (E3BP) (54 kDa, sc-79236, Santa Cruz Biotechnology), purified goat polyclonal anti-UCP2 (35 kDa) (sc-6525, Santa Cruz Biotechnology), purified goat polyclonal anti-mitochondrial superoxide dismutase (SOD2, 25 kDa) (sc-18503, Santa Cruz Biotechnology), rabbit polyclonal anti-citrate synthetase (CS, 52 kDa) (ab-96600, Abcam), mouse monoclonal anti-mitochondrial marker (MTC02, 60 kDa), and the MitoProfile® total OXPHOS human antibody cocktail (MS601, MitoScience) containing antibodies raised against subunit of Complex I (20 kDa subunit NDUFB8), Complex II (30 kDa subunit), Complex III (subunit Core 2, 47 kDa), Complex IV (COXII, 24 kDa) and ATP synthase (subunit α, 57 kDa). Appropriate horseradish peroxidase-conjugated secondary antibodies were used. The expression levels of COXII or mitochondrial marker (for the mitochondrial fractions) and of β-actin (for the cell fractions) were used as loading normalisation controls. Protein bands were visualised using the Amersham ECL system and digitally quantified using the GeneTools 4.03 software package.

### Statistical analysis

The results are presented as the means ± SE obtained from at least three to eight independent experiments (cell suspension preparations or mitochondrial isolations), and each determination was performed at least in duplicate throughout this study. An unpaired two-tailed Student’s *t*-test was used to identify significant differences; in particular, differences were considered to be statistically significant if *p* < 0.01 (*), *p* < 0.05 (**), or *p* < 0.001 (***).

## Results

### Measurements of mitochondrial respiratory function in EA.hy926 cells and the Crabtree effect

To assess mitochondrial bioenergetics in endothelial cells, the optimal number of detached EA.hy926 cells and the equivalent concentration of total cell protein needed to obtain a measurable OCR were established. Cellular oxygen consumption was proportional to cell concentration; in particular, the range of 10 × 10^6^–40 × 10^6^ cells/ml was equivalent to 1–4 mg of protein per ml, independent of the respiratory substrates (Fig. S2a). For subsequent experiments, a cell protein concentration of 2 mg/ml (equivalent to approximately 20 × 10^6^ cells/ml) was selected for the optimal detection of changes in the OCR of cells. Because the detachment of cells may result in anoikis, which is associated with increased ROS and mitochondrial damage, the measurements were all performed within 4 h; during this period, cell viability is retained [15].

To determine the time-course of respiratory response induced by excess glucose, we measured basal OCR of EA.hy926 cells after 3, 6, and 9 days of treatment with 5.5 or 25 mM glucose (Fig. 1). A statistically significant decrease in basal OCR was observed in cells grown in high glucose for at least 6 days or longer. These measurements indicate that the respiratory response of endothelial cells to glucose depends on the duration of exposure to elevated glucose concentrations. Therefore, further experiments were performed with EA.hy926 cells grown for 6 days in normal or high glucose.

**Fig. 2:**
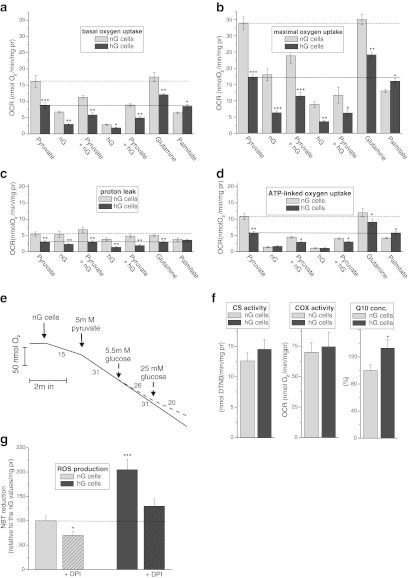
Mitochondrial function of EA.hy926 cells grown in normal-glucose concentration (*nG* cells) or high-glucose concentration (*hG* cells). Substrate-dependent changes in the basal oxygen consumption rate (*OCR*) (**a**), maximal OCR (**b**), proton leak (**c**), and ATP-dependent OCR (**d**). Substrates: 5 mM pyruvate, 5.5 mM glucose (nG), 25 mM glucose (hG), pyruvate and nG, pyruvate and hG, 3 mM l-glutamine, or 0.3 mM palmitate. The *vertical lines* illustrate the values in the presence of pyruvate alone in nG cells (*dashed lines*) and hG cells (*solid lines*). **e** The representative oxygen uptake measurement with EA.hy926 cells (using nG cells as an example), illustrating the Crabtree effect. The numbers on the traces refer to the OCR in nmol O/min/mg protein. **f** The COX activity, CS activity, and cellular concentration of *Q*_10_ (expressed as the percentage of Q10 concentration in nG cells). **g** NBT reduction in the absence or presence of 10 μM DPI

To examine how EA.hy926 cells grown in normal or high glucose respond to simple change in respiratory substrates, mitochondrial respiratory function was measured with the following substrates: pyruvate alone; 5.5 mM glucose alone; 25 mM glucose alone; a combination of pyruvate and either 5.5 mM or 25 mM glucose; glutamine; or palmitate. Under basal conditions (basal OCR) (Fig. 2a), FCCP-stimulated conditions (maximal OCR) (Fig. 2b), and the presence of oligomycin (the oligomycin-resistant OCR, ATP-linked OCR) (Fig. 2d), both types of cells demonstrated the highest OCR with pyruvate alone or glutamine and the lowest OCR with 25 mM glucose alone. However, in the cells grown in high glucose, the OCR with glutamine was significantly higher than the OCR with pyruvate, whereas in the cells grown at normal glucose, both of these substrates were oxidised at a similar level. Moreover, in the high-glucose cells, the OCR with palmitate was similar to the OCR with pyruvate, whereas in the normal-glucose cells, the OCR with palmitate was much lower than the OCR with pyruvate. These results indicate an increased oxidation of glutamine and palmitate in the cells cultured under high-glucose conditions. Among the tested OCRs, the proton leak (non-ATP-linked OCR) exhibited the least dependence on the type of substrate that was applied (Fig. 2c).

For both types of EA.hy926 cells, the highest (at least 5-fold) difference between respiration with pyruvate as the sole substrate and respiration with 25 mM glucose alone was observed in the ATP-linked OCR and the basal OCR (Fig. 2). In general, for all of the examined conditions, glucose decreased the OCR of both types of cells in a concentration-dependent manner. The Crabtree effect was also observed during the sequential addition of glucose to the medium of pyruvate-oxidising cells under basal conditions (Fig. 2e), as well as during glutamine oxidation (data not shown).

### In endothelial cells, growth in high-glucose concentrations caused lowered mitochondrial respiration during carbohydrate and glutamine oxidation and increased respiration with fatty acids

In general, under all conditions and with respect to all substrates except palmitate, the EA.hy926 cells grown at high-glucose concentrations displayed significantly lowered mitochondrial function relative to the cells exposed to normal-glucose concentrations (Fig. 2). In particular, the high-glucose cells exhibited at least 2-fold lower maximal mitochondrial respiratory capacity with pyruvate alone, glucose alone (either 5.5 or 25 mM), or any combination of these substrates (Fig. 2b); for glutamine oxidation, a less dramatic decrease (only approximately 1.4-fold decrease) in the maximal OCR was observed. By contrast, the oxidation of palmitate was significantly higher in the high-glucose cells, and similar results were observed using stearic acid, another free fatty acid (data not shown). These results indicate that there is a greater contribution from fatty acids as fuels of endothelial respiration under high-glucose conditions.

Interestingly, no significant differences in ATP-linked OCR were observed between both types of cells during the oxidation of glucose alone (either 5.5 or 25 mM), indicating similar low levels of mitochondrial oxidative phosphorylation during carbohydrate catabolism (Fig. 2d). The apparent mitochondrial reserve capacity (maximal OCR/basal OCR) did not differ significantly between both types of cells for any of the tested substrates (Supplementary Data).

**Fig. 3:**
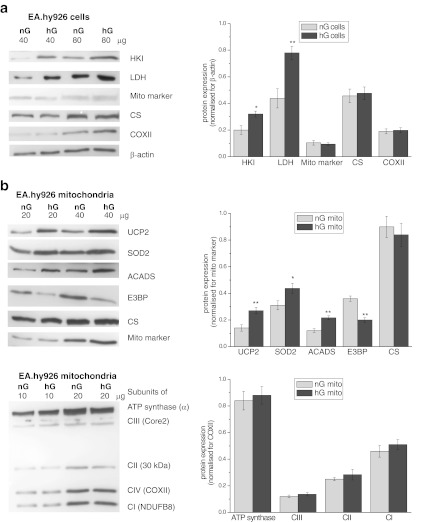
Determination of protein levels in EA.hy926 cells grown in normal glucose (*nG* cells) or high glucose (*hG* cells) (**a**) and in mitochondria isolated from these cells (nG mito and hG mito, respectively) (**b**). **a** Representative Western blots and analyses of the protein expression of HKI, LDH, mitochondrial marker (Mito marker), CS, and COXII. **b** Representative Western blots and analyses of the protein expression of UCP2, SOD2, ACADS, E3BP, CS, and particular subunits of ATP synthase, Complex III (*CIII*), Complex II (*CII*) and Complex I (*CI*). Expression levels normalised for β actin (**a**), mito marker (**b** *, upper panel*), or COXII (**b** *, lower panel*) protein abundance are shown

The endothelial cells cultured in normal or high-glucose conditions exhibited similar CS and COX activities, indicating that the different growth conditions did not change the capacity of the TCA cycle or the mitochondrial respiratory chain (Fig. 2f). Moreover, no differences in the expressions of mitochondrial marker, COXII, and CS were detected (Fig. 3a). However, a significant upregulation of hexokinase I (HKI) and LDH was observed in the cells grown in high glucose (Fig. 3a), indicating that these cells displayed intensified anaerobic glucose oxidation through a glycolic pathway and lactate fermentation. Additionally, a significantly greater cellular Q10 content was found in the cells grown under high-glucose conditions.

### High glucose-induced increase of ROS generation

As shown in Fig. 2g, compared with cells cultured in normal glucose conditions, exposure of EA.hy926 cells to high-glucose concentrations caused 2-fold increase of ROS generation. In addition, we found that DPI, a flavoprotein inhibitor of NADPH oxidase, inhibited significantly hyperglycaemia-induced ROS generation. However, DPI-insensitive ROS generation (mitochondrial ROS generation) was significantly higher in the high-glucose cells. Therefore, in EA.hy926 cells hyperglycaemia-induced ROS appears to be produced through the enzyme NADPH oxidase and from mitochondrial sources.

### Glycolytic endothelial cells possess active, well-coupled mitochondria

**Fig. 4:**
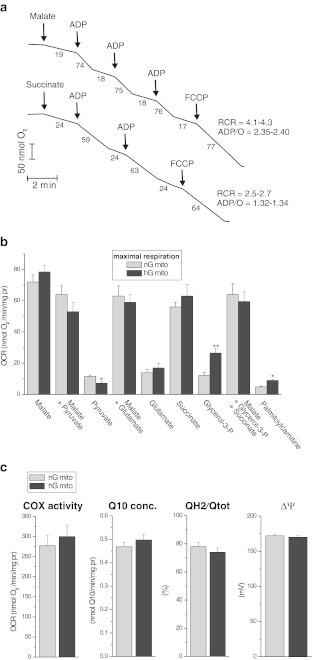
Functional characteristics of endothelial mitochondria isolated from normal-glucose and high-glucose cells (nG mito and hG mito, respectively). **a** Representative oxygen uptake measurements (using nG mitochondria as an example) of non-phosphorylating respiration, phosphorylating respiration, and uncoupled respiration, as well as the obtained coupling parameters, ADP/O and the respiratory control ratio (*RCR*). Malate (10 mM) and 10 mM succinate (plus 1 μM rotenone) were used as respiratory substrates. The numbers on the traces refer to OCR in nmol O/min/mg protein. **b** The maximal respiration (phosphorylating respiration or uncoupled respiration) with different respiratory substrates: 10 mM malate, 10 mM pyruvate, 10 mM glutamate, 10 mM succinate, 3 mM glycerol-3-phosphate, 0.3 mM palmitoyl*-* dl *-*carnitine*.* **c** The COX activity, concentration of *Q*_10_ in mitochondria, and mitochondrial *Q* reduction level (QH_2_/*Q*_tot_) and the membrane potential (Δ*Ψ*) during non-phosphorylating oxidation of succinate

Our efficient procedure of mitochondrial isolation from cultured EA.hy926 cells produced highly active and well-coupled mitochondria (Fig. 4a). The mitochondria isolated from both types of cells exhibited good coupling parameters, i.e., high ADP/O and respiratory control ratios with malate or succinate. These results indicate that mitochondrial electron transport through the respiratory chain of isolated endothelial mitochondria is coupled well with ATP synthesis when Complex I and Complex II substrates are applied. Moreover, the mitochondria were quite stable for 6–7 h and their outer mitochondrial membrane exhibited good integrity (97–99 %).

The highest maximal mitochondrial respiration (phosphorylating or uncoupled respiration) was observed with malate, either alone or supplemented with pyruvate, glutamate or a combination of succinate and glycerol-3-phospate, and with succinate alone (Fig. 4b). Malate oxidation appeared to saturate the capacity of the mitochondrial respiratory chain, as the addition of the other reducing substrate(s) to malate did not further increase mitochondrial respiration. Interestingly, the apparent maximal mitochondrial respiration comprised approximately 27 % of the apparent capacity of Complex IV (COX) (Fig. 4c). Glutamate (an intermediate of amino acid metabolism), glycerol-3-phosphate and palmitoylcarnitine (lipid breakdown intermediates) were more weakly oxidised by EA.hy926 mitochondria than were malate or succinate (TCA cycle intermediates). Interestingly, the oxidation of pyruvate alone was not very intense compared with the oxidation of these two TCA cycle substrates.

Growth in high glucose induced an increase in mitochondrial oxidation of palmitoylcarnitine and glycerol-3-phosphate and a decrease in pyruvate oxidation, whereas the TCA cycle and respiratory chain remained unaffected

Growth under high-glucose concentrations did not influence the activity or composition of basic respiratory chain components in EA.hy926 mitochondria. The cytochrome pathway activity, COX activity, and oxidative phosphorylation efficiency did not change, and the mitochondrial Δ*Ψ* and *Q* reduction level of the resting state (with succinate as the oxidisable substrate) were also unaffected. Moreover, the expression levels of subunits of ATP synthase (α) and the four respiratory chain complexes, namely, Complex I (NDUFB8), Complex II (subunit 30 kDa), Complex III (Core2), and Complex IV (COXII) (Fig. 3b), as well as the Q10 content of the mitochondria (Fig. 4c), were not affected by high-glucose growth. In addition, no change in the expression level or activity of CS was observed (Fig. 3a, b and f), indicating that high-glucose conditions produced no discernible change at the level of the TCA cycle.

However, in response to high-glucose conditions, the upregulation of the expression of mitochondrial antioxidant proteins, such as superoxide dismutase (SOD2) and UCP2, was observed (Fig. 3b). Moreover, in EA.hy926 mitochondria, a high glucose level increased the expression of acyl-CoA dehydrogenase (ACADS), which catalyses the initial step of fatty acid β-oxidation. This finding is consistent with the greater oxidation of palmitoylcarnitine and glycerol-3-phosphate that was observed in mitochondria isolated from the high-glucose cells (Fig. 4b), indicating the greater oxidation of reducing substrates not originating from the TCA cycle. By contrast, these mitochondria demonstrated significantly decreased expression of the E3BP component of the pyruvate dehydrogenase complex (Fig. 3b) and significantly decreased pyruvate oxidation relative to mitochondria from cells cultured under normal glucose levels (Fig. 4b).

### High-glucose conditions induced increased UCP2 activity

**Fig. 5:**
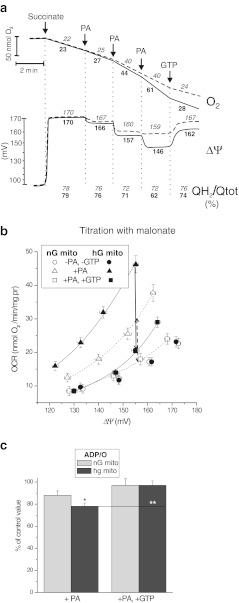
UCP2 activity in endothelial mitochondria from normal-glucose and high-glucose cells. **a** Effects of UCP2 activation by palmitic acid (PA) (7 μM per addition), and UCP2 inhibition by 2 mM GTP on the oxygen uptake, Δ*Ψ*, and *Q* reduction level. The normal-glucose mitochondria (nG mito) are represented by *broken lines* and numbers in *italics*, and the high-glucose mitochondria (hG mito) are represented by *solid* lines and *bold* numbers. **b** Relationship between the respiratory rate and Δ*Ψ* (proton leak kinetics) during non-phosphorylating succinate oxidation titrated with malonate. The PA-induced, GTP-inhibited, UCP2-mediated proton leak at the same Δ*Ψ* (155 mV) is indicated as *vertical lines* (a *broken line* indicates nG mitochondria, and a *solid line* indicates hG mitochondria). **c** The influence of PA and GTP on the ADP/O ratio during the phosphorylating respiration. Relative changes compared with the control values (in the absence of PA and GTP) are shown. **b**, **c** Succinate (with 1 μM rotenone) was used as and oxidisable substrate. The mitochondria were incubated in the absence or presence of 14 μM PA and/or 2 mM GTP

To determine whether UCP2 is functionally active in endothelial mitochondria, we evaluated the activation of UCP2 by free fatty acids and the inhibition by GTP in isolated EA.hy926 mitochondria. In non-phosphorylating mitochondria isolated from cells grown in high-glucose conditions, the maximal rate of the respiration induced by palmitic acid, which presented UCP2 activity, was 2-fold higher (Fig. 5a). Similarly, a 2-fold greater decrease in the Δ*Ψ* and *Q* reduction level was observed upon maximal activation of UCP2 by the fatty acid. Similar effects were observed using another free fatty acid, linoleic acid (data not shown). The addition of 2 mM GTP partially reversed the changes induced by palmitic acid in both types of mitochondria. Moreover, a proton leak kinetics study (Fig. 5b) indicates that for given palmitic acid (14 μM) and GTP (2 mM) concentrations, the palmitic-acid-induced, GTP-inhibited, UCP2-mediated proton leak at the same Δ*Ψ* (155 mV) was also 2-fold higher in the mitochondria from the high-glucose cells. Under phosphorylating conditions, the increased activity of UCP2 in the mitochondria from the high-glucose cells led to a significantly greater reduction in the oxidative phosphorylation yield (Fig. 5c). For given palmitic acid (14 μM) and GTP (2 mM) concentrations, the palmitic-acid-induced, GTP-reversed drop in the ADP/O ratio was almost 2-fold greater in the mitochondria from the high-glucose cells than in the mitochondria from the normal-glucose cells.

## Discussion

The comparison between the mitochondrial respiratory functions of EA.hy926 cells cultured in medium with either high or normal glucose demonstrated that chronic high-glucose conditions induced a reduction in mitochondrial respiration with carbohydrate catabolism intermediates (glucose, pyruvate, or a combination of both of these substrates) and glutamine, whereas OCR with palmitate (an intermediate in lipid metabolism) was increased. The Crabtree effect was also observed in both types of cells when we studied the influence of 5.5 or 25 mM glucose on pyruvate oxidation. In the cells grown in high glucose, the OCR with glutamine was significantly higher than the OCR with pyruvate. These results indicate that hyperglycaemia lowers the contribution of carbohydrate oxidation to the aerobic metabolism of EA.hy926 cells, whereas the contributions of amino acid oxidation and lipid oxidation increase. Interestingly, it has been shown previously that in human umbilical vein endothelial cells (HUVECs), fatty acids can serve as an important energy source; moreover, the activation of the fuel-sensing enzyme AMPK favours the oxidation of fatty acids as the source of ATP production and reduces dependence on glycolysis [3]. In addition, the high-glucose EA.hy926 cells examined in this study seem to undergo a shift of aerobic metabolism towards the oxidation of lipids that resembles the change in the myocardial energy utilisation that is induced by high glucose levels. In particular, diabetes is associated with a switch in myocardial substrate utilisation that results in decreased glucose oxidation and increased fatty acid oxidation [26]. Our results show for the first time that a shift to a predominant oxidation of fatty acids which accompanies diabetic changes in metabolism could also occur in endothelial cells. Manipulation of proteins that are upregulated in endothelial cells under high glucose conditions (LDH, HKI, UCP2, SOD2) by overexpression or siRNA could pinpoint in the future the molecules responsible for the described switch in oxidative metabolism.

In EA.hy926 cells grown for 6 days in high glucose, the Crabtree effect (the decreased OCR in the presence of glucose) was accompanied by the increased expression of both HKI, the enzyme catalysing the first step, which is the rate-limiting step, of the glycolytic pathway, and LDH, the enzyme that catalyses the interconversion of pyruvate and lactate. These results indicate the increased anaerobic and decreased aerobic (mitochondrial) breakdown of glucose in EA.hy926 cells grown under chronic high-glucose conditions. Similarly, it has been shown that HUVECs and bovine retinal endothelial cells (BRECs) grown in high glucose for 7 days exhibit elevated lactate production [14]. Interestingly, a short (up to 45 min) exposure of human microvascular endothelial cells (HMECs) to 25 mM glucose appears to produce no change in lactate production or OCR, although increased levels of a glycolytic intermediate, glucose 6-phosphate, have been observed [24]. Therefore, it appears that the respiratory response of endothelial cells to high glucose depends on the period of the exposure to elevated glucose (Fig. 1). A decrease in respiration was observed in EA.hy926 cells grown in high glucose for at least 6 days or longer. Therefore, the present work describing a chronic exposure to elevated glucose does not reflect the effect of a short-term hyperglycaemia on endothelial cells.

The exposure of endothelial cells to high glucose levels leads to increased intracellular and mitochondrial ROS production and therefore produces excessive oxidative stress [2, 8, 12, 17, 18, 23, 29, 31, 32]. Under our experimental conditions, the increased oxidative stress in EA.hy926 cells grown under chronic high-glucose conditions was revealed by significantly higher intracellular and mitochondrial ROS generation and upregulation of the expression of mitochondrial antioxidative system proteins, such as SOD2 and UCP2. The increased expression levels of SOD2 and UCP2 in response to high glucose have previously been observed for other endothelial cells [2, 32]. Mitochondrial contribution to hyperglycaemia-induced ROS production was also observed in BAECs, BRECs, HUVECs and retinal capillary endothelial cells, including cells chronically exposed to elevated glucose [2, 8, 18, 29, 31, 32]. In EA.hy926 endothelial cells, hyperglycaemia-induced ROS appears to be produced through the enzyme NADPH oxidase and from mitochondrial sources. However, our results indicate that glucose-fuelled oxidative metabolism cannot rather mediate oxidative stress because OCR with carbohydrate catabolism intermediates (glucose, pyruvate, or a combination of both of these substrates) was impaired in the EA.hy926 cells grown in high glucose. Moreover, mitochondria isolated from the high-glucose cells demonstrated significantly decreases in both pyruvate oxidation and the expression of the E3BP component of the pyruvate dehydrogenase complex. Thus, it appears that the supply of energy to the TCA cycle from pyruvate, a key intermediate in several metabolic pathways, is impaired in EA.hy926 cells grown in high glucose. This finding is consistent with previously promulgated suggestions that the accumulation of glucose 6-phosphate during the incubation of endothelial cells under high-glucose concentrations indicates that downstream metabolic steps are rate-limiting [24].

The greater contribution of lipid oxidation to the aerobic metabolism of the EA.hy926 cells grown in high glucose is indicated by the increased expression of ACADS and the higher oxidation of palmitoylcarnitine and glycerol-3-phosphate in the mitochondria that were isolated from these cells. Glycerol-3-phosphate serves as a major link between carbohydrate metabolism and lipid metabolism, and the glycerol-3-phosphate shuttle is an important electron supply to the animal mitochondrial respiratory chain. Thus, the increased level of reducing substrates originating from lipid metabolism likely mediates the endothelial oxidative stress induced by excess glucose. In general, mitochondrial ROS generation is associated with increased levels of reduction of respiratory chain components (Complex I and III) that may be due to the increased oxidation of mitochondrial fuels and/or the impairment of the QH_2_-oxidising pathway [27]. The impaired production of nitric oxide (NO, a competitive inhibitor of Complex IV, COX) observed in endothelial cells exposed to acute or chronic high glucose levels [7, 23] does not account for the latter possibility. However, nitric oxide synthase III (NOSIII) as being a source of ROS through other mechanism cannot be excluded under our experimental conditions. The operation of the Crabtree effect would decrease the contribution of ROS produced by mitochondria and favour the contributions from other steps of glucose metabolism in the cell, including via increased NADPH oxidase activity [24]. Thus, under high-glucose conditions, endothelial mitochondria could stimulate the overall intracellular production of ROS, despite the fact these mitochondria may not generally be a major source of ROS [4]. In high-glucose EA.hy926 cells, a higher cellular content of *Q*_10_ (in contrast to the mitochondrial *Q*_10_ content) may indicate an increased need for this lipid-soluble antioxidant, given that an elevated level of *Q*_10_ has been observed to be a protective response to excessive oxidative stress in certain diseases, including endothelial dysfunction [16].

Recently, measurements of mitochondrial function through extracellular flux analysis in adherent primary BAECs have indicated that endothelial mitochondria are highly coupled to ATP synthesis and possess a considerable bioenergetic reserve, although exposure to sublethal oxidative stress reduces this reserve capacity [6]. In EA.hy926 cells, the apparent mitochondrial reserve capacity did not differ for any of the tested substrates between cells grown in normal and high glucose. This study provided the first measurement of endothelial mitochondrial function, directly demonstrating that in isolated EA.hy926 mitochondria, electron transport is coupled well with ATP synthesis. Thus, endothelial cells that are primarily glycolytic possess active, well-coupled mitochondria, despite the fact that these mitochondria do not generate significant contributions to cellular ATP production through oxidative phosphorylation.

Our results indicate that EA.hy926 mitochondria possessed UCP2 activity that was stimulated by free fatty acids and inhibited by purine nucleotides; moreover, this UCP2 activity was 2-fold higher in mitochondria from cells cultured in high glucose. The high-glucose-induced increase in the activity of UCP2 led to a greater reduction in the oxidative phosphorylation yield, indicating that one physiological role of UCP2 would be the attenuation of mitochondrial ROS production under oxidative stress conditions, particularly given that endothelial mitochondria are not particularly dependent on oxidative phosphorylation. Our results confirm the implication that UCP2 may serve as a sensor and a negative regulator of mitochondrial ROS production in endothelial cells with elevated glucose levels [2, 18, 29, 32].

We can conclude that although the synthesis of ATP in endothelial cells occurs primarily via a glycolytic pathway [4, 5], these cells possess highly active and well-coupled mitochondria with a functioning energy-dissipating pathway that involves UCP2. The growth of endothelial cells under high-glucose conditions induces numerous changes in the cells’ aerobic metabolism, particularly with respect to the Crabtree effect, as well as a shift towards the oxidation of lipids and amino acids. These results indicate the role of endothelial mitochondria in response to metabolic disturbances that relate to hyperglycaemia.

## Electronic supplementary material

Below is the link to the electronic supplementary material.

ESM 1 (DOC 915 kb)
