# Supplementary material for: The influence of high glucose on the aerobic metabolism of endothelial EA.hy926 cells
Source: Pflugers Arch. 2012 Sep 30;464(6):657–69. doi: 10.1007/s00424-012-1156-1 (PMC3513600; doi:10.1007/s00424-012-1156-1)
Supplement: Supplementary file 1 — (DOC 915 kb) [file 424_2012_1156_MOESM1_ESM.doc]

**SUPPLEMENTARY DATA**

**Citrate synthase activity**

CS activity was essentially assayed as described by Freitas *et al.* (2010) [1]. Suspensions of detached cells were placed in SET buffer (0.32 M sucrose, 1 mM EDTA, 10 mM Tris-HCl, pH 7.4) (1-1.2 mg pr/ ml) and homogenised with Polytron (3 x 2 sec). All of the steps were performed at 4^o^C. After a short (30 sec) centrifugation of unbroken cells and cell debris, the supernatant was collected for the determination of CS activity. The reaction mixture (1 ml) contained 100 μM Tris, pH 8.0; 100 μM acetyl-Co; 100 mM 5,5’-di-thiobis-(2-nitrobenzoic acid) (TNB); 0.1% triton X-100; and 40-60 μg supernatant protein, and was initiated with 100 μM oxaloacetate and monitored at 412 nm for 3 min at 37^o^C.

**References**

1. Freitas TP, Rezin GT, Gonçalves CL, Jeremias GC, Gomes LM, Scaini G, Teodorak BP, Valvassori SS, Quevedo J, Streck (2010) Evaluation of citrate synthase activity in brain of rats submitted to an animal model of mania induced by ouabain. Mol Cell Biochem 341: 245-249

**
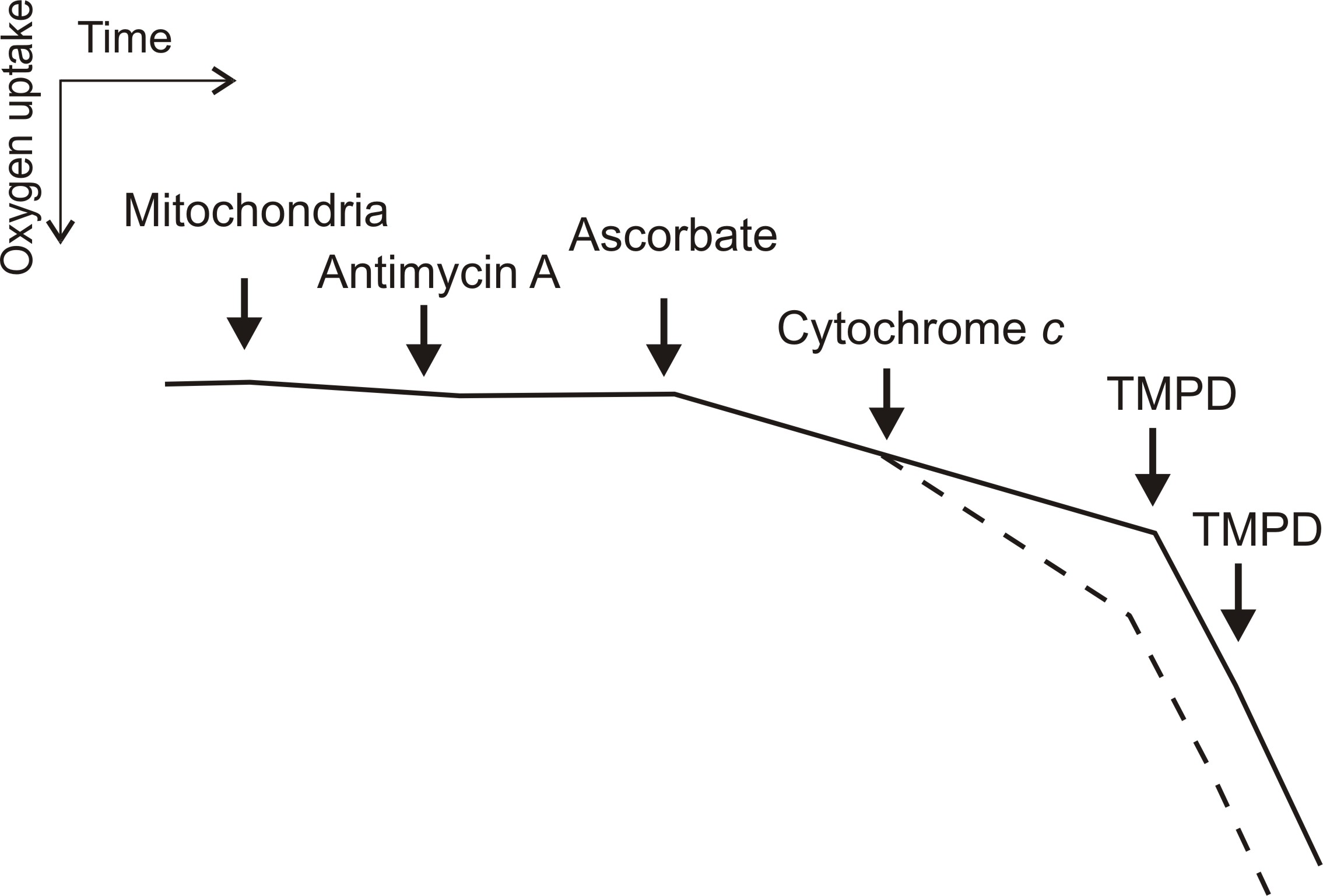
**

**Supplementary Fig. 1** Assessment of the cytochrome *c* oxidase (COX) maximal activity and testing the integrity of the outer mitochondrial membrane. COX maximal activity was assessed with 0.25 mg of mitochondrial protein without exogenously added respiratory substrate and in the presence of sequentially added antimycin A (10 μM), 8 mM ascorbate, 0.06% cytochrome *c*, and up to 2 mM N,N,N’N’-tetramethyl-*p*-phenylenediamine (TMPD). The rate of oxygen consumption following the addition of TMPD reflected the maximal O_2_ consumption by COX. Exogenous cytochrome *c* was added to assess the outer membrane integrity. An increase in oxygen consumption with exogenous cytochrome *c* is apparent if the outer mitochondrial membrane is damaged (a broken line). A full line illustrates a preparation where membrane integrity is high. The integrity was calculated from oxygen consumption rates (OCRs) in the presence of given chemicals using the following equation: [TMPD_OCR_ – cytochrome *c* _OCR_]/ [TMPD_OCR_ – ascorbate _OCR_] x 100%.

**
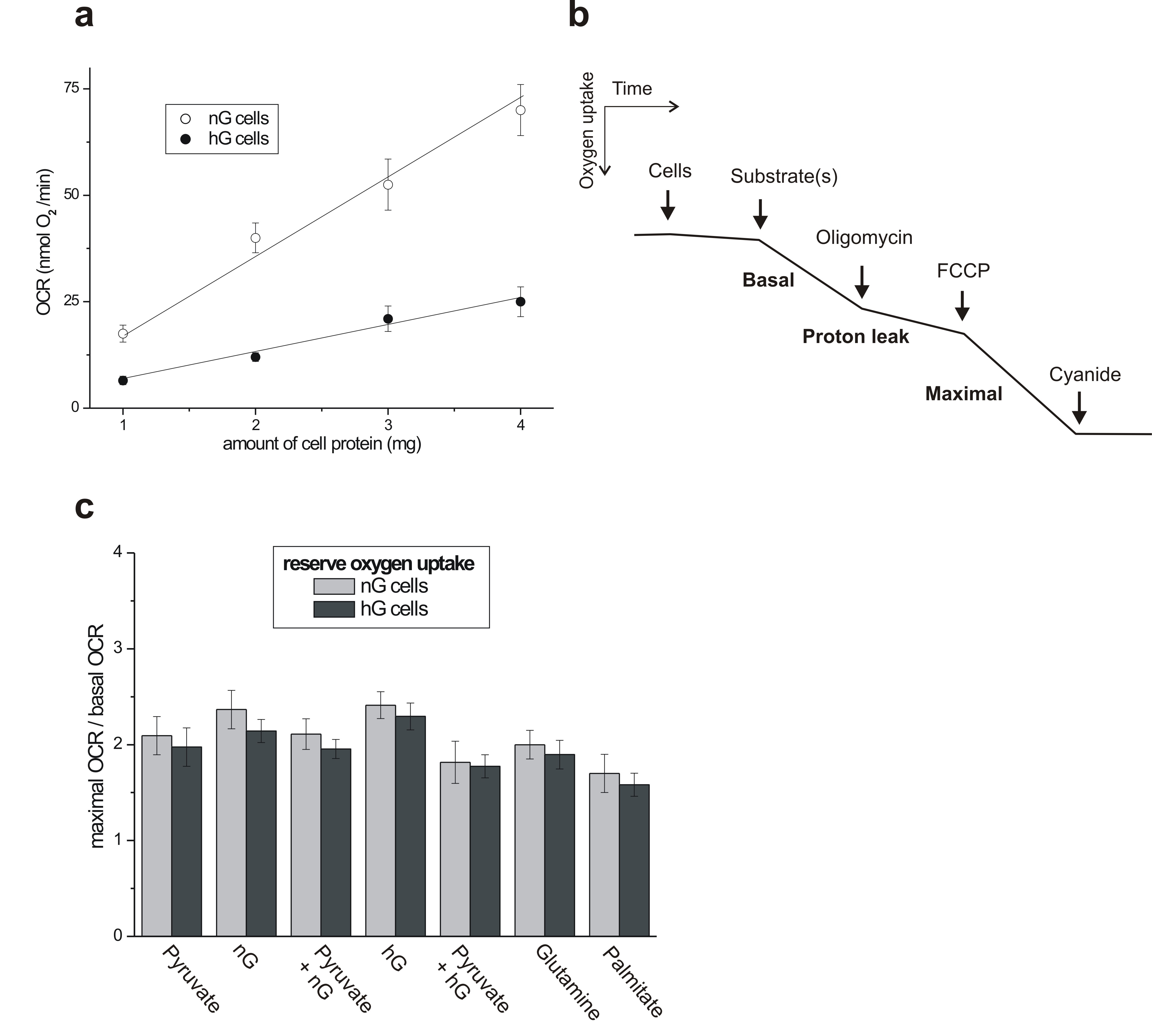
**

**Supplementary Fig. 2** Measurement of mitochondrial function in EA.hy926 cells grown in normal-glucose (nG cells) or high-glucose (hG cells) media. (**a**) The basal oxygen consumption rate (OCR) plotted as a function of the amount of cell protein. The data are the means ± S.E. (*n* = 9) for three independent cell suspensions. (**b**) A scheme for the OCR measurements with the sequential addition of respiratory substrate(s), oligomycin (1 μg/ml), FCCP (0.4 μM), and cyanide (0.5 mM). The basal OCR was measured in the presence of substrate(s) only. The proton leak (the non-ATP-linked OCR) corresponds to the oligomycin-resistant OCR. The ATP-linked OCR was calculated as the difference between the basal OCR and the oligomycin-resistant OCR. The maximal OCR was measured after the addition of the ionophore, FCCP. No residual oxygen consumption, and thus no non-mitochondrial respiration, was observed in the presence of cyanide, which contrasts with the result obtained if antimycin A (10 μM) was used (data not shown). (**c**) The apparent reserve OCR (the maximal OCR/basal OCR) for different oxidisable substrates: 5 mM pyruvate, 5.5 mM glucose (nG), 25 mM glucose (hG), pyruvate and nG, pyruvate and hG, 3 mM L-glutamine, or 0.3 mM palmitate.
